# *N*-(2-Chloro-5-methyl­phen­yl)succinamic acid

**DOI:** 10.1107/S1600536811054638

**Published:** 2011-12-23

**Authors:** B. Thimme Gowda, Sabine Foro, U. Chaithanya

**Affiliations:** a Department of Chemistry, Mangalore University, Mangalagangotri 574 199, Mangalore, India; b Institute of Materials Science, Darmstadt University of Technology, Petersenstrasse 23, D-64287 Darmstadt, Germany

## Abstract

In the title compound, C_11_H_12_ClNO_3_, the conformation of the N—H bond in the amide segment is *syn* with respect to the *ortho*-Cl atom. The amide and carboxyl C=O groups are *syn* to each other. Furthermore, the C=O and O—H bonds of the carboxyl group are in *syn* positions with respect to each other. The dihedral angle between the benzene ring and the amide group is 47.8 (2)°. In the crystal, mol­ecules are connected by pairs of O—H⋯O hydrogen bonds, forming inversion dimers. The dimers are further linked by N—H⋯O hydrogen bonds into double chains along the *b*-axis direction.

## Related literature

For our previous studies on the effects of substituents on the structures and other aspects of *N*-(ar­yl)-amides, see: Gowda *et al.* (2001 ▶); Saraswathi *et al.* (2011 ▶), on *N*-(ar­yl)-methane­sulfonamides, see: Jayalakshmi & Gowda (2004 ▶), on *N*-(ar­yl)-aryl­sulfonamides, see: Gowda *et al.* (2005 ▶) and on *N*-chloro­aryl­amides, see: Gowda *et al.* (1996 ▶). For modes of hydrogen bonding in the structures of carb­oxy­lic acids, see: Leiserowitz (1976 ▶). For the centrosymmetrical dimeric hydrogen-bonding association of carb­oxy­lic groups, see: Jagannathan *et al.* (1994 ▶).
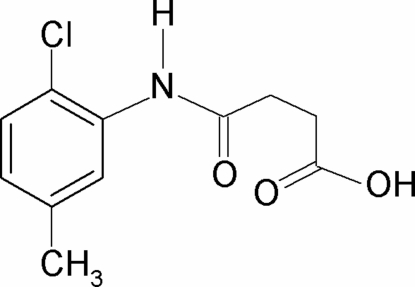


## Experimental

### 

#### Crystal data

C_11_H_12_ClNO_3_
*M* *_r_* = 241.67
Monoclinic, 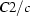

*a* = 23.780 (5) Å
*b* = 4.7784 (7) Å
*c* = 23.892 (5) Å
β = 121.20 (1)°
*V* = 2322.2 (8) Å^3^
*Z* = 8
Mo *K*α radiation
μ = 0.32 mm^−1^
*T* = 293 K
0.42 × 0.10 × 0.08 mm

#### Data collection

Oxford Diffraction Xcalibur with Sapphire CCD detector diffractometer
Absorption correction: multi-scan (*CrysAlis RED*; Oxford Diffraction, 2009 ▶) *T*_min_ = 0.877, *T*_max_ = 0.975
4399 measured reflections
2322 independent reflections
1680 reflections with *I* > 2σ(*I*)
*R*_int_ = 0.020

#### Refinement

*R*[*F* ^2^ > 2σ(*F* ^2^)] = 0.062
*wR*(*F* ^2^) = 0.128
*S* = 1.19
2322 reflections
152 parameters
2 restraints
H atoms treated by a mixture of independent and constrained refinement
Δρ_max_ = 0.28 e Å^−3^
Δρ_min_ = −0.25 e Å^−3^

### 

Data collection: *CrysAlis CCD* (Oxford Diffraction, 2009 ▶); cell refinement: *CrysAlis CCD*; data reduction: *CrysAlis RED* (Oxford Diffraction, 2009 ▶); program(s) used to solve structure: *SHELXS97* (Sheldrick, 2008 ▶); program(s) used to refine structure: *SHELXL97* (Sheldrick, 2008 ▶); molecular graphics: *PLATON* (Spek, 2009 ▶); software used to prepare material for publication: *SHELXL97*.

## Supplementary Material

Crystal structure: contains datablock(s) I, global. DOI: 10.1107/S1600536811054638/yk2035sup1.cif

Structure factors: contains datablock(s) I. DOI: 10.1107/S1600536811054638/yk2035Isup2.hkl

Supplementary material file. DOI: 10.1107/S1600536811054638/yk2035Isup3.cml

Additional supplementary materials:  crystallographic information; 3D view; checkCIF report

## Figures and Tables

**Table 1:** Hydrogen-bond geometry (Å, °)

*D*—H⋯*A*	*D*—H	H⋯*A*	*D*⋯*A*	*D*—H⋯*A*
O3—H3*O*⋯O2^i^	0.83 (2)	1.83 (2)	2.652 (4)	172 (5)
N1—H1*N*⋯O1^ii^	0.86 (2)	2.08 (2)	2.910 (4)	163 (3)

Symmetry codes: (i) 
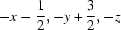
; (ii) 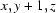.

**Fig. 1.**
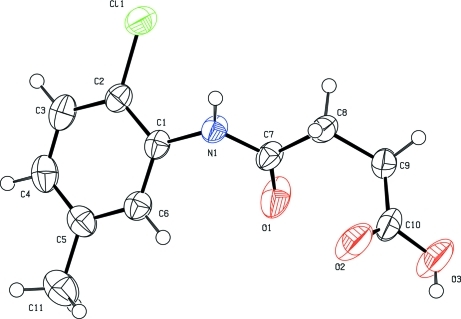
Molecular structure of the title compound, showing the atom labelling scheme and with displacement ellipsoids drawn at the 50% probability level.

**Fig. 2.**
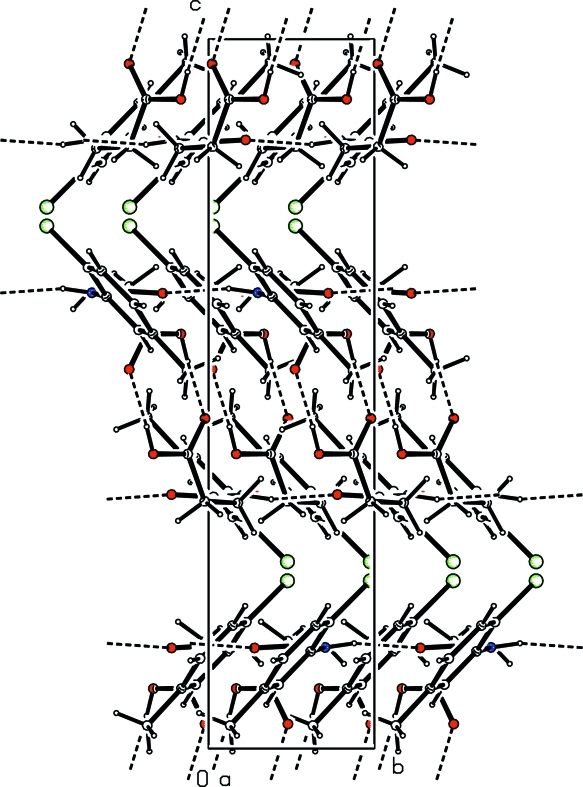
Molecular packing of the title compound with hydrogen bonds shown as dashed lines.

## supplementary crystallographic information

### Comment

The amide and sulfonamide moieties are important constituents of many biologically important compounds. As a part of our studies of the substituent effects on the structures and other aspects of *N*-(aryl)-amides (Gowda *et al.*, 2001; Saraswathi *et al.*, 2011), *N*-(aryl)-methanesulfonamides (Jayalakshmi & Gowda, 2004), *N*-(aryl)-arylsulfonamides (Gowda *et al.*, 2005) and *N*-chloro-arylsulfonamides (Gowda *et al.*, 1996), in the present work, the crystal structure of *N*-(2-chloro-5-methylphenyl)succinamic acid (I) has been determined (Fig. 1). The conformation of the N—H bond in the amide segment is *syn* to the *ortho*–chloro group and *anti* to the *meta*–methyl group in the benzene ring, similar to the *syn* conformation observed between the amide hydrogen and the *ortho*-Cl and *anti* conformation between the amide hydrogen and the *meta*-Cl in the benzene ring of *N*-(2,5-dichlorophenyl)- succinamic acid (II) (Saraswathi *et al.*, 2011).

Further, the conformations of the amide oxygen and the carboxyl oxygen of the acid segment are *syn* to each other.

The C═O and O—H bonds of the acid group are in *syn* position to each other, similar to that observed in (II).

The dihedral angle between the phenyl ring and the amide group is 47.8 (2)°.

The intermolecular O—H···O and N—H···O hydrogen bonds pack the molecules into infinite chains along the *b*-axis direction (Table 1, Fig.2).

The modes of interlinking carboxylic acids by hydrogen bonds is described elsewhere (Leiserowitz, 1976). The packing of molecules involving dimeric hydrogen-bonded association of each carboxyl group with a centrosymmetrically related neighbor has also been observed (Jagannathan *et al.*, 1994).

### Experimental

The solution of succinic anhydride (0.01 mole) in toluene (25 ml) was treated with the solution of 2-chloro,5-methylaniline (0.01 mole) also in toluene (20 ml), added dropwise with permanent stirring. The resulting mixture was stirred for about one hour and set aside for an additional hour at room temperature for completion of the reaction. The mixture was then treated with diluted hydrochloric acid to remove the unreacted 2-chloro-5-methyl-aniline. The resultant title compound was filtered under suction and washed thoroughly with water to remove the unreacted succinic anhydride and succinic acid. The product was recrystallized to constant melting point from ethanol. The purity of the compound was checked and characterized by its infrared and NMR spectra.

Rod like colorless single crystals used in X-ray diffraction studies were grown from ethanolic solution by slow evaporation at room temperature.

### Refinement

The H atoms of the NH and OH groups were located in a difference map and later restrained to the distances N—H = 0.86 (2) Å and O—H = 0.82 (2) Å, respectively. The other H atoms were positioned with idealized geometry and refined using a riding model with the aromatic C—H = 0.93 Å and methylene C—H = 0.97 Å. All H atoms were refined with isotropic displacement parameters (set to 1.2 times of the *U*_eq_ of the parent atom).

### Crystal data

**Table:**

C_11_H_12_ClNO_3_	*F*(000) = 1008
*M**_r_* = 241.67	*D*_x_ = 1.382 Mg m^−^^3^
Monoclinic, *C*2/*c*	Mo *K*α radiation, λ = 0.71073 Å
Hall symbol: -C 2yc	Cell parameters from 1604 reflections
*a* = 23.780 (5) Å	θ = 2.6–27.9°
*b* = 4.7784 (7) Å	µ = 0.32 mm^−^^1^
*c* = 23.892 (5) Å	*T* = 293 K
β = 121.20 (1)°	Rod, colourless
*V* = 2322.2 (8) Å^3^	0.42 × 0.10 × 0.08 mm
*Z* = 8	

### Data collection

**Table:**

Oxford Diffraction Xcalibur with Sapphire CCD detector diffractometer	2322 independent reflections
Radiation source: fine-focus sealed tube	1680 reflections with *I* > 2σ(*I*)
graphite	*R*_int_ = 0.020
ω and φ scans	θ_max_ = 26.4°, θ_min_ = 3.4°
Absorption correction: multi-scan (*CrysAlis RED*; Oxford Diffraction, 2009)	*h* = −29→28
*T*_min_ = 0.877, *T*_max_ = 0.975	*k* = −4→5
4399 measured reflections	*l* = −20→29

### Refinement

**Table:**

Refinement on *F*^2^	Primary atom site location: structure-invariant direct methods
Least-squares matrix: full	Secondary atom site location: difference Fourier map
*R*[*F*^2^ > 2σ(*F*^2^)] = 0.062	Hydrogen site location: inferred from neighbouring sites
*wR*(*F*^2^) = 0.128	H atoms treated by a mixture of independent and constrained refinement
*S* = 1.19	*w* = 1/[σ^2^(*F*_o_^2^) + (0.0268*P*)^2^ + 6.3165*P*] where *P* = (*F*_o_^2^ + 2*F*_c_^2^)/3
2322 reflections	(Δ/σ)_max_ = 0.044
152 parameters	Δρ_max_ = 0.28 e Å^−^^3^
2 restraints	Δρ_min_ = −0.25 e Å^−^^3^

### Special details

**Table:**

Geometry. All s.u.'s (except the s.u. in the dihedral angle between two l.s. planes) are estimated using the full covariance matrix. The cell s.u.'s are taken into account individually in the estimation of s.u.'s in distances, angles and torsion angles; correlations between s.u.'s in cell parameters are only used when they are defined by crystal symmetry. An approximate (isotropic) treatment of cell s.u.'s is used for estimating s.u.'s involving l.s. planes.
Refinement. Refinement of *F*^2^ against ALL reflections. The weighted *R*-factor *wR* and goodness of fit *S* are based on *F*^2^, conventional *R*-factors *R* are based on *F*, with *F* set to zero for negative *F*^2^. The threshold expression of *F*^2^ > σ(*F*^2^) is used only for calculating *R*-factors(gt) *etc*. and is not relevant to the choice of reflections for refinement. *R*-factors based on *F*^2^ are statistically about twice as large as those based on *F*, and *R*- factors based on ALL data will be even larger.

### Fractional atomic coordinates and isotropic or equivalent isotropic displacement parameters (Å^2^)

**Table:**

	*x*	*y*	*z*	*U*_iso_*/*U*_eq_	
Cl1	0.14042 (4)	1.47549 (19)	0.23661 (4)	0.0481 (3)	
O1	−0.02815 (14)	0.7770 (5)	0.14193 (17)	0.0703 (9)	
O2	−0.18862 (14)	0.9786 (7)	0.03462 (14)	0.0749 (9)	
O3	−0.21661 (15)	0.6674 (7)	0.08465 (14)	0.0783 (10)	
H3O	−0.2438 (19)	0.623 (10)	0.0462 (12)	0.094*	
N1	0.01311 (13)	1.2030 (5)	0.14261 (14)	0.0363 (6)	
H1N	0.0095 (16)	1.378 (4)	0.1481 (16)	0.044*	
C1	0.06702 (14)	1.1156 (6)	0.13712 (15)	0.0330 (7)	
C2	0.12917 (15)	1.2274 (7)	0.17838 (15)	0.0362 (7)	
C3	0.18210 (16)	1.1446 (8)	0.17339 (18)	0.0473 (9)	
H3	0.2235	1.2205	0.2011	0.057*	
C4	0.17314 (17)	0.9488 (8)	0.12704 (18)	0.0487 (9)	
H4	0.2090	0.8916	0.1243	0.058*	
C5	0.11180 (17)	0.8360 (7)	0.08458 (17)	0.0423 (8)	
C6	0.05926 (15)	0.9222 (7)	0.09027 (16)	0.0377 (8)	
H6	0.0177	0.8484	0.0620	0.045*	
C7	−0.03027 (15)	1.0299 (7)	0.14512 (16)	0.0377 (7)	
C8	−0.08153 (15)	1.1768 (7)	0.15378 (18)	0.0418 (8)	
H8A	−0.1036	1.3159	0.1196	0.050*	
H8B	−0.0599	1.2737	0.1955	0.050*	
C9	−0.13225 (16)	0.9767 (8)	0.15140 (17)	0.0452 (8)	
H9A	−0.1096	0.8164	0.1788	0.054*	
H9B	−0.1553	1.0697	0.1698	0.054*	
C10	−0.18136 (16)	0.8754 (8)	0.08466 (19)	0.0450 (9)	
C11	0.1020 (2)	0.6300 (8)	0.03259 (19)	0.0605 (11)	
H11A	0.1074	0.4431	0.0494	0.073*	
H11B	0.1339	0.6646	0.0201	0.073*	
H11C	0.0586	0.6509	−0.0049	0.073*	

### Atomic displacement parameters (Å^2^)

**Table:**

	*U*^11^	*U*^22^	*U*^33^	*U*^12^	*U*^13^	*U*^23^
Cl1	0.0457 (5)	0.0473 (5)	0.0465 (5)	−0.0107 (4)	0.0206 (4)	−0.0069 (4)
O1	0.0751 (19)	0.0230 (14)	0.147 (3)	−0.0019 (13)	0.081 (2)	−0.0023 (16)
O2	0.0717 (18)	0.081 (2)	0.0607 (17)	−0.0415 (17)	0.0259 (15)	−0.0011 (17)
O3	0.072 (2)	0.084 (2)	0.0667 (19)	−0.0466 (18)	0.0269 (16)	−0.0004 (18)
N1	0.0339 (13)	0.0231 (13)	0.0559 (17)	−0.0001 (12)	0.0261 (13)	−0.0033 (13)
C1	0.0331 (16)	0.0245 (16)	0.0432 (18)	0.0027 (13)	0.0210 (14)	0.0072 (14)
C2	0.0366 (16)	0.0327 (18)	0.0381 (17)	−0.0027 (14)	0.0185 (14)	0.0039 (15)
C3	0.0334 (17)	0.053 (2)	0.055 (2)	−0.0001 (16)	0.0220 (16)	0.0081 (19)
C4	0.0430 (19)	0.052 (2)	0.061 (2)	0.0101 (18)	0.0338 (18)	0.012 (2)
C5	0.056 (2)	0.0349 (19)	0.0450 (19)	0.0061 (16)	0.0326 (17)	0.0065 (16)
C6	0.0382 (17)	0.0322 (18)	0.0430 (18)	0.0004 (14)	0.0212 (14)	0.0030 (15)
C7	0.0319 (16)	0.0283 (18)	0.0519 (19)	−0.0012 (14)	0.0210 (14)	0.0002 (16)
C8	0.0342 (16)	0.0336 (19)	0.059 (2)	−0.0058 (15)	0.0254 (16)	−0.0103 (17)
C9	0.0421 (18)	0.045 (2)	0.060 (2)	−0.0081 (17)	0.0346 (17)	−0.0092 (18)
C10	0.0332 (17)	0.040 (2)	0.065 (2)	−0.0062 (15)	0.0281 (17)	0.0000 (18)
C11	0.080 (3)	0.052 (2)	0.064 (3)	0.011 (2)	0.047 (2)	0.004 (2)

### Geometric parameters (Å, °)

**Table:**

Cl1—C2	1.740 (3)	C4—H4	0.9300
O1—C7	1.214 (4)	C5—C6	1.387 (4)
O2—C10	1.220 (4)	C5—C11	1.506 (5)
O3—C10	1.300 (4)	C6—H6	0.9300
O3—H3O	0.832 (19)	C7—C8	1.511 (4)
N1—C7	1.347 (4)	C8—C9	1.517 (4)
N1—C1	1.418 (4)	C8—H8A	0.9700
N1—H1N	0.858 (18)	C8—H8B	0.9700
C1—C6	1.388 (4)	C9—C10	1.487 (5)
C1—C2	1.390 (4)	C9—H9A	0.9700
C2—C3	1.382 (4)	C9—H9B	0.9700
C3—C4	1.379 (5)	C11—H11A	0.9600
C3—H3	0.9300	C11—H11B	0.9600
C4—C5	1.384 (5)	C11—H11C	0.9600
			
C10—O3—H3O	109 (4)	O1—C7—C8	122.3 (3)
C7—N1—C1	125.0 (3)	N1—C7—C8	114.3 (3)
C7—N1—H1N	117 (2)	C7—C8—C9	112.6 (3)
C1—N1—H1N	118 (2)	C7—C8—H8A	109.1
C6—C1—C2	118.4 (3)	C9—C8—H8A	109.1
C6—C1—N1	121.5 (3)	C7—C8—H8B	109.1
C2—C1—N1	120.1 (3)	C9—C8—H8B	109.1
C3—C2—C1	120.7 (3)	H8A—C8—H8B	107.8
C3—C2—Cl1	119.6 (3)	C10—C9—C8	114.4 (3)
C1—C2—Cl1	119.7 (2)	C10—C9—H9A	108.7
C4—C3—C2	119.6 (3)	C8—C9—H9A	108.7
C4—C3—H3	120.2	C10—C9—H9B	108.7
C2—C3—H3	120.2	C8—C9—H9B	108.7
C3—C4—C5	121.2 (3)	H9A—C9—H9B	107.6
C3—C4—H4	119.4	O2—C10—O3	123.0 (4)
C5—C4—H4	119.4	O2—C10—C9	123.6 (3)
C4—C5—C6	118.3 (3)	O3—C10—C9	113.4 (3)
C4—C5—C11	120.9 (3)	C5—C11—H11A	109.5
C6—C5—C11	120.9 (3)	C5—C11—H11B	109.5
C5—C6—C1	121.8 (3)	H11A—C11—H11B	109.5
C5—C6—H6	119.1	C5—C11—H11C	109.5
C1—C6—H6	119.1	H11A—C11—H11C	109.5
O1—C7—N1	123.4 (3)	H11B—C11—H11C	109.5
			
C7—N1—C1—C6	49.1 (5)	C4—C5—C6—C1	−0.2 (5)
C7—N1—C1—C2	−132.0 (3)	C11—C5—C6—C1	−178.8 (3)
C6—C1—C2—C3	−0.9 (5)	C2—C1—C6—C5	1.0 (5)
N1—C1—C2—C3	−179.8 (3)	N1—C1—C6—C5	179.9 (3)
C6—C1—C2—Cl1	178.8 (2)	C1—N1—C7—O1	−1.1 (6)
N1—C1—C2—Cl1	−0.2 (4)	C1—N1—C7—C8	177.6 (3)
C1—C2—C3—C4	−0.1 (5)	O1—C7—C8—C9	−5.4 (5)
Cl1—C2—C3—C4	−179.7 (3)	N1—C7—C8—C9	175.8 (3)
C2—C3—C4—C5	1.0 (5)	C7—C8—C9—C10	−75.1 (4)
C3—C4—C5—C6	−0.8 (5)	C8—C9—C10—O2	−12.7 (5)
C3—C4—C5—C11	177.8 (3)	C8—C9—C10—O3	168.1 (3)

### Hydrogen-bond geometry (Å, °)

**Table:**

*D*—H···*A*	*D*—H	H···*A*	*D*···*A*	*D*—H···*A*
O3—H3O···O2^i^	0.83 (2)	1.83 (2)	2.652 (4)	172 (5)
N1—H1N···O1^ii^	0.86 (2)	2.08 (2)	2.910 (4)	163 (3)

Symmetry codes: (i) −*x*−1/2, −*y*+3/2, −*z*; (ii) *x*, *y*+1, *z*.
